# Physical Activity Guidance Resources for Rural Families of Neurodiverse or Developmentally Diverse Children: Exploratory Co-Design Study

**DOI:** 10.2196/92658

**Published:** 2026-07-14

**Authors:** Kate Freire, Rodney Pope, Kristen Andrews, Jason Bennie, Megan Suthern

**Affiliations:** 1 Three Rivers Department of Rural Health, Charles Sturt University, Elizabeth Mitchell Drive, Thurgoona, New South Wales, 2640, Australia, 61 260519361, 61 1800 275278; 2 School of Allied Health, Exercise and Sports Sciences, Charles Sturt University, Thurgoona, New South Wales, Australia; 3 Murrumbidgee Primary Health Network, Wagga Wagga, NSW, Australia; 4 Paediatric Department, Wagga Wagga Base Hospital, Wagga Wagga, New South Wales, Australia

**Keywords:** co-design, physical activity, family, neurodiversity, parenting

## Abstract

**Background:**

Being active benefits children and their parents, but there is a lack of co-designed resources that support families to engage in physical activity (PA) together.

**Objective:**

This exploratory co-design study aimed to identify ways to optimize guidance resources intended to assist in being active together, and to evaluate the co-design approach it used.

**Methods:**

Physiotherapist-researchers with experience and expertise in family PA drafted 2 evidence-informed prototypes. One prototype informed families about the holistic benefits of being active together, while the other helped families to plan their episode of PA. Twelve parents from 9 rural families and their neurodiverse or developmentally diverse children (n=13) were recruited as co-designers to ensure a wide range of considerations. Family co-designers took part in 2 to 3 workshops. They trialed the prototypes, telling researchers what worked well and how to improve them. Child and adult advisory groups oversaw the co-design approach. Each group met 3 times, with the child advisory group meeting prior to the adult advisory group. A child voice facilitator and advocate was used to support the child advisory groups and facilitate communication between child and adult advisory groups.

**Results:**

Sixty-two changes were made to the prototypes in response to feedback from families and researcher observations. Key changes to resources recommended by children, parents, and researchers to increase user acceptability and usability included changing the ordering of the planning resource to reflect the way that most families used it, adding a “*hard words*” section to improve readability, support to normalize that no-one wins all the time, additional examples to cover each section of the resources, and encourage negotiation among families to ensure that the planned PA was mutually desirable. All families used the resources to successfully complete, or adapt, their planned session of PA together. Overall, co-designers and advisory group members perceived the co-design approach to be appropriate and responsive. However, the researchers identified a possible gap in final communication with the children regarding dissemination of results of the project which were provided to families via parents’ email.

**Conclusions:**

The co-design approach used in this study supported families, including neurodiverse and developmentally diverse children, to trial and provide feedback on PA resources. The large number of changes made to the resources from co-designer suggestions and researcher observations highlights the importance of gaining user feedback for consumer resources. Future research is needed to evaluate how these co-designed resources may impact family PA behavior and identity. The transparent co-design approach and evaluation highlighted limitations for research communication to children, which require careful consideration in future similar studies.

## Introduction

The widespread benefits from regular physical activity (PA) are dose related, with the greatest health benefits observed among those who go from inactive to active [1,2]. National and international guidelines for PA emphasize minimum recommendations for PA volume and intensity [3-5]. Yet, despite decades of public health messaging on PA, regular child PA participation remains low worldwide [6]. For example, a 2022 report from the Active Healthy Kids Global Alliance graded 10 indicators related to PA across 57 countries. Aggregate grades include D for overall PA grade, C+ for PA engagement at school, and C− for family and peer PA engagement [6]. Low child PA participation rates are concerning, given the widespread health and well-being benefits childhood PA affords children throughout their childhood and adult life [7,8]. Children who are neurodiverse and developmentally diverse are at an even greater risk of inactivity due in part to slower development of fundamental movement and prosocial skills [9-11]. These populations include children with conditions such as autism, attention deficit hyperactivity disorder, and developmental coordination disorder, as well as those who are not meeting their developmental milestones. Diagnosis of conditions takes time, and many families, particularly those in rural areas, endure long waiting lists to access developmental assessments [12].

Parents shape their children’s PA behaviors, which underlines the importance of supporting their endeavors. For example, medium-sized correlations have been found in studies of parental support behaviors and child PA [13,14]. There is general consensus in the literature that the following parental behaviors assist child PA: encouragement and talking about PA, logistical support, and cross-generational PA (X-gen PA; ie, parents or carers being active with their children) [13]. In 2022, Active Healthy Kids Australia suggested increasing child and parent engagement in PA *together* to provide modeling opportunities for children and reduce risks of chronic diseases (ie, diabetes) while increasing both populations’ opportunities for a healthy life [15].

Research has found that X-gen PA provides more than physical and psychological health benefits [16]. For example, X-gen PA has been identified by both children and parents as an important instrument through which they can connect to each other socially and emotionally [16]. It facilitates communication, in a nonconfrontational context, and provides a bonding experience, even in contexts where verbal communication is not feasible (eg, high-intensity activity), possibly via mirroring of similar repetitive movements [16]. Parents view the support they provide during X-gen PA as much broader than just assisting their child’s PA [16]. For example, they use it to teach their children how to work with other people and build resilience by getting through physically challenging circumstances together, as well as to role model healthy behavior and help their children to gain and practice sporting skills [16].

X-gen PA is a reciprocal partnership to which children, as well as parents, contribute [17,18]. Children may influence the commencement of an episode of PA with their parents, which requires consensus from all those involved [18]. For example, children may initiate episodes of PA with their parents by bringing their siblings with them to augment their request or ask to join in their parent’s PA session [18]. It is evident that X-gen PA is a complex bidirectional phenomenon when viewed through the lens of socioecological models, such as those described by Bronfenbrenner [19] and James and Prout [20]. Yet, X-gen PA is often considered in PA research through a unidirectional model as one aspect of parental support for child PA [21,22]. This unidirectional model, where children’s influence is not acknowledged and parents are considered the sole *gatekeepers* of family PA, may have contributed to the lack of children’s voices in family PA research [18] and the narrow focus of PA promotion by targeting parents only [23].

A recent systematic review identified a lack of co-designed holistic resources that support families to engage in PA *together*, including among families with neurodiverse and developmentally diverse children [23]. This oversight is surprising as co-designing with end users ensures that resources are suitable for the populations they are targeting. In addition, co-designing with end users is recognized as one way of increasing health equity and literacy [24].

Family-centered practice describes another socioecological model that supports health professionals and researchers to work in partnership with families to enhance developmental outcomes for children [25]. It is strengths-based, acknowledging that all families have strengths that can be built upon, including that they are the experts on their *own* family [1,26]. Lundy’s model of participation [27] was developed to help operationalize Article 12 of the Charter on the Rights of the Child [28], which states that children have a right to be heard on issues that affect them. The model describes 4 chronological elements that should be considered to support children’s participation: space, voice, audience, and influence [27].

Children living in rural areas in Australia exhibit greater developmental vulnerability. For example, an overview of the 2021 Australian Early Developmental Census identified that only 55% of children in the geographic setting for this study were developmentally on track across all 5 domains: physical health and well-being, social competence, emotional maturity, language and cognitive skills (school-based), and communication skills and general knowledge [29].

The primary aim of the research was to identify ways to improve the usability and acceptability of guidance resources intended to increase knowledge of how to plan and engage in X-gen PA among rural families, including those with children who are neurodiverse and developmentally diverse. The secondary aim was to explore the authenticity of the co-design approach by evaluating the approach through the voices of children and parent co-designers, child and adult advisory groups, and researchers.

## Methods

### Overview

**Figure 1.**
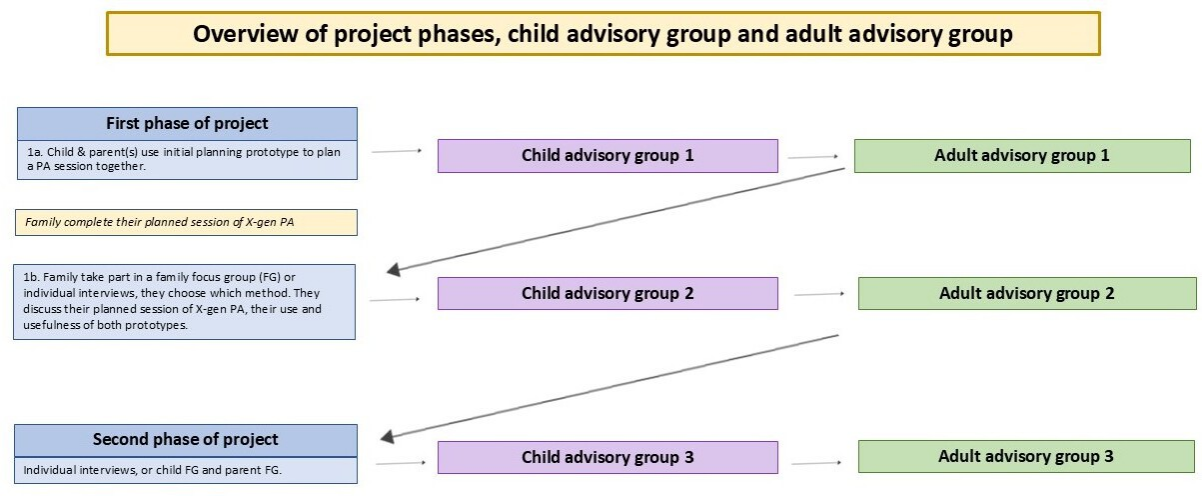
Co-design phases and advisory groups.

The research was conducted within a critical realism paradigm, which acknowledges both the existence of objective reality (ontological realism) and that co-designers’ and researchers’ understanding of that reality is situated and relative (epistemological relativism) [30]. The study used an exploratory, sequential, participatory research design [31,32] using multiple methods to iteratively inform resource co-design. The co-design process involved physiotherapist-researchers with experience and expertise in X-gen PA, along with neurodiverse or developmentally diverse children and the parents of these children. The physiotherapist-researchers contributed to the co-design process knowledge of considerations regarding X-gen PA gleaned from families more broadly based on prior research with families [16,18] with their own families’ lived experiences with X-gen PA and their professional experiences. Participation and contributions of neurodiverse and developmentally diverse children and their parents in the co-design process enabled consideration of a range of additional issues for X-gen PA that may not have been as prevalent or as strongly recognized in many of the families involved in prior X-gen PA research. Children and parent co-designers attended 2 to 3 co-designer workshops where they trialed and provided feedback and suggestions on key elements of 2 X-gen PA prototype resources that were initially drafted by the physiotherapist-researchers, based on their prior understanding of X-gen PA (Figure 1). Additionally, child and adult advisory groups were established to provide guidance and feedback on the co-design approach (Figure 1). Both quantitative data (from surveys of participants) and qualitative data (child and parent co-designer suggestions and researcher observations) from workshops were collected and analyzed in this multimethod co-design research.

### Setting

This study took place in a rural Local Health District (LHD) in NSW, Australia. Co-design workshops and child advisory group meetings were held in local library meeting rooms, while the adult advisory group meetings were online.

### Drafting of Evidence-Informed Prototype X-Gen PA Guidance Resources

As a first step in the sequential co-design process undertaken in this study, 2 evidence-informed prototype X-gen PA guidance resources were drafted by the physiotherapist-researchers (KF, KA, and RP) in the research team. These drafts provided families participating in the co-design study with evidence-based prototypes to trial and critique, thereby facilitating a sequential approach to co-design in which the initial contributions of the practitioner-researchers, resulting in prototypes, provided a foundation for the contributions of children and parents and subsequent contributions of the researchers. Similar sequential approaches to co-design, which integrate research evidence and clinical and expert experience with lived experiences of consumers through a sequential co-design process, have been described elsewhere, for example, the Framework for Co-Design of Clinical Practice Tools [31]. A further example is the bidirectional strategy used in the co-design of a self-compassion mobile health intervention for people with cancer, which enabled the merging of “top-down” (research evidence based and clinical expertise based) and “bottom-up” (lived experience based) inputs in the co-design process [32].

In this study, the drafted prototypes took the form of booklets that provided families with guidance on building family capacity for planning and adapting PA to the X-gen PA context. Drafting of the prototypes was informed by evidence accumulated from previous research [16,18] and the following frameworks and behavior change theories: the International Classification of Functioning, Disability, and Health [33], family-centered practice [25], self-determination theory [33], socioecological frameworks [20], and strengths-based practice [1].

The first of these prototype resources, *Doing Physical Activity Together,* described the purposes behind X-gen PA and experiences of children and parents with X-gen PA, discussed current evidence of the benefits of being active together, and provided examples of types of PA that families might try doing together. The second prototype resource, *Planning Physical Activity Together,* covered aspects families might consider when planning a PA session together. It also guided families through a postsession review, through which they could identify aspects of the X-gen PA session that each participant liked and aspects they might wish to change in future PA sessions.

The prototype resources sought to assist autonomous experiences by supporting children’s and parents’ rights to take part in planning and choosing aspects of X-gen PA [34]. Competence was addressed in the *Planning Physical Activity Together* prototype [33], which stepped co-designers through every aspect of planning an episode of PA together. In addition, the prototypes provided families with a common language to plan and discuss their X-gen PA. It was anticipated that this planning process would enable children to be active participants in decision-making and that the resources would build capacity for planning and adapting PA across both generations, which would be mutually reinforcing. Three quotes from children and parents involved in preceding X-gen PA research and suggestions from that prior research for X-gen PA were included in *You might like to try* sections of the prototypes to provide ideas for families and promote norms for X-gen PA. Two Australian First Nations mothers were paid as consultants to examine the initial proposed text in the 2 X-gen resource prototypes and provide feedback on their cultural appropriateness. No changes were identified as being needed by the 2 Australian First Nations mothers. This suggested that the prototypes were culturally safe for use by Australian First Nations families, should they choose to participate in the co-design process.

Child and parent co-designers subsequently commenced engagement with the prototype resources by using the planning resource to plan a session of PA together. This approach allowed all co-designers, irrespective of their experience of X-gen PA, to take part in the co-design study, as there was no requirement for previous experience of X-gen PA. Further details are provided below of the extensive involvement and contributions of children and parents in the subsequent stages of the co-design process.

### Researchers, Partner Organizations, and Child Voice Facilitator and Advocate

Two researchers (KF and KA) facilitated all co-design workshops involving children and parents. Both were experienced physiotherapists, with more than two decades of experience working with children and families, as well as experience conducting research workshops and focus groups and with X-gen PA. The study involved a partnership with the Pediatric Department of an LHD, a private rural pediatric clinic, and a primary health network. The partners assisted with recruitment and provided advice for the project through representatives in project planning and on the adult advisory group. An experienced primary school learning support officer was recruited into the role of child voice facilitator and advocate (CVFA). The CVFA was a dual role created to provide both independent (nonresearcher) facilitatory support to the child advisory group and an advocacy role for the child’s voice at adult advisory group meetings.

### Child and Parent Co-Designers

Purposive sampling was used to invite and secure the participation of a diverse group of families who would likely use the co-designed resources. Pediatricians identified neurodiverse or developmentally diverse children aged between 5 and 12 years who also met other eligibility criteria for participation. The parents and carers of these children were subsequently informed about the study, and both the parents (or carers) and children were invited to participate. Exclusion criteria were those with a health condition that severely limited PA participation and family members who required an interpreter to take part. Parent consent and child assent for participation were obtained.

To acknowledge their time and expertise, child co-designers were given a suitable piece of PA equipment, and parent co-designers and CVFA received a credit card voucher following participation in each workshop or advisory group.

### Co-Design Workshops

The co-design research approach was chosen to ensure that both child and parent voices, reflecting their lived experiences, contributed to the final X-gen PA guidance resources, together with the evidence-based understandings and lived experiences of X-gen PA among the researchers. Lundy’s model of participation, encompassing the 4 elements of *space, voice, audience,* and *influence*, was used to inform the co-design research approach [27]. The checklist developed by Ireland’s Department of Children and Youth Affairs [35] was used by the researchers to aid reflexivity on all elements of the approach (Multimedia Appendix 1).

Data collection periods involving participating children and families were determined by co-designers’ preferences and availability and occurred during the first 3 school holiday periods in 2024 (Table 1), with phase 1 occurring in January, phase 2 in April, and the final child and adult advisory group meetings (Table 2) occurring during the July 2024 holiday period.

**Table 1.** Data collection and activities with participating children and parents.

Study phase and activity	Participants	Description
Phase 1: January school holidays		
Initial questionnaires	Child and parent co-designers	Children completed a paper questionnaire about intergenerational physical activity (X-gen PA^a^), with researcher assistance where appropriate. Parents completed a paper questionnaire on X-gen PA and demographic information (eg, age, gender, and health conditions).
Workshop 1: planning	Child and parent co-designers working together	Co-designers provided feedback while working through the Planning Physical Activity Together prototype. Researchers observed and documented suggestions for improvement. Families left with their completed plan, both prototypes, and an agreed date for the second interview. Families completed their planned physical activity session together.
Workshop 2: reflecting	Child and parent co-designers working together	The session began with opportunities for co-designers to reflect on their experiences of completing the plan through drawing and writing activities. Beginning with child participants, co-designers described their physical activity session and provided further critique of the planning prototype and initial feedback on the Doing Physical Activity Together prototype.
Phase 2: April school holidays		
Workshop 3: doing	Child and parent co-designers separately	Children played a board game in which landing on a specific color corresponded to a section of the Doing Physical Activity Together prototype. Colored cards contained questions about each section. Parents reviewed the prototype one page at a time, suggested improvements, identified gaps, and highlighted aspects they liked.
Post–X-gen PA questionnaires	Child and parent co-designers	Children completed a paper questionnaire, with researcher assistance where appropriate. Parents completed a paper questionnaire.
Evaluation of co-design	Child and parent co-designers	Participants completed questionnaires based on the Lundy rights-compliant model [27]. The child questionnaire was adapted from the evaluation questionnaire in the Government of Ireland Participation Framework for Children and Young People Participation in Decision Making [36]. Parents completed a corresponding questionnaire.

a X-gen PA: cross-generational physical activity.

**Table 2.** Summary of child and adult advisory group meetings.

Meetings	Child advisory group	Adult advisory group
Meeting 1	Introductions and game. Discussion with researchers covering: What do child advisors do? What is research? Why should research hear from children? How would you like to tell us what you think? Brainstorming and discussion on how child advocates can support the group. How do the children want the child advocate to inform the adult advisory group what they think?	Introductions. Presentation of project. Co-design approach and evaluation. Draft terms of reference for the adult advisory group. Briefing on the child advisory group from the child advocate. Agree feedback to child advisory group (sent to child advisory group members via parent email). Feedback invited from group on project.
Meeting 2	Introductions and game. Review adult advisory group feedback. Briefing on first phase of project. Watch video of researchers explaining some of the changes made to resource in response to child and adult advice. Score and provide feedback on how well researchers have listened. Brainstorm what children want adult advisory group to know.	Introduction and attendance. Review terms of reference of group. Briefing and feedback invited on co-design during first phase of project. Briefing on child advisory group from child advocate. Agree feedback to child advisory group (sent to child advisory group members via parent email). Discuss co-design approach in phase 1.
Meeting 3	Introductions and game. Review adult advisory group feedback. Researcher invited into group to remind group and discuss why hearing from children is important. Briefing on second phase of project. Watch video of researchers explaining some of the changes made to resource in response to child and adult advice. Score and provide feedback on how well researchers have listened. Brainstorm what children want adult advisory group to know. Present children with participation certificates, thanking them for being child advisors.	Introduction and attendance. Review terms of reference of group. Briefing and feedback invited on co-design second phase of project. Briefing on child advisory group from child advocate. Agree feedback to child advisory group (sent to child advisory group members via parent email). Where to from here? Brainstorming translation of project. Group invited to contribute to evaluation of co-design approach.

Parents completed a short demographic survey at the beginning of workshop 1. The child and parent co-designers used the initial planning prototype *Planning Physical Activity Together* to plan a session of PA together, while a researcher observed and provided advice where appropriate. Child and parent co-designers left with copies of both prototypes. They returned following the implementation of their plan for an X-gen PA session. Achievements reported by the child and parent co-designers in completing X-gen plans were recorded, along with their initial experiences of using the resources and impacts they reported the resources had on their understandings of planning and doing PA together. Child and parent co-designers then provided feedback and gave their suggestions on the 2 prototypes. In phase 2, separate concurrent child and parent workshops were conducted, and co-designers were asked, as part of a small post–co-design survey, if using the resources had changed the ways they (parents and children) were active together and to evaluate the co-design approach (Table 1).

### Child and Adult Advisory Groups

All child and parent co-designers were invited to join either the child or adult advisory group, respectively, to oversee the project and provide advice on the co-design approach (Table 2). Three child advisory group meetings were held during the research period. Adult advisory group meetings were held after each child advisory group meeting. Child advisory group meetings were facilitated by the CVFA (with online support from a project officer). The CVFA also attended adult advisory group meetings in their advocacy capacity, telling the adult advisory group what the child advisors wanted them to know and providing independent, nonresearcher representations of the child’s voice, as requested by the children in the child advisory group. The CVFA reported back from the adult group to the child group via the children’s parents’ email addresses. Adult advisory group meetings included a parent co-designer, the CVFA, a pediatrician, a data support officer from the primary health network, and the physiotherapist-researchers involved in the project.

Advisory group members were invited, during the second and third advisory group meetings, to comment on changes made to the prototype resources in response to data gathered in the co-design process. Child advisors watched a short video of how researchers had responded to changes suggested by child co-designers. The CVFA invited child advisors to then score how well the changes matched what child co-designers had told researchers, using a 5-point smile rating scale. They were then invited to say why they had given those scores.

Adult advisory group members were provided with tables, which listed *all* comments from all co-designers and all observations from researchers, together with the results of initial data analysis and any related changes made to the prototype resources. Subsequently, a brief overview of key changes made to the prototypes was provided during adult advisory group meetings before the advisory group members were invited to comment and ask questions. Adult advisory group members (excluding the physiotherapist-researchers) were invited to evaluate the co-design approach via a survey after the last advisory group meeting.

### Reporting of Co-Design Approach

A reporting checklist was used to ensure that there was comprehensive reporting of the co-design approach while noting that exact replication is not an aim in the reporting of participatory approaches because they should be adapted to the specific context [23] (Multimedia Appendix 2).

### Data Analysis

All co-design workshops were audio recorded and transcribed. All co-designers’ suggestions and researchers’ observations were tabulated and counted. Researchers met regularly during the data collection period to discuss all suggestions and observations, reaching a consensus on amendments to be made to the prototype resources in response to this feedback. Conventional content analysis (as described by Hsieh and Shannon [37]) was used to group suggestions and observations of all co-designers into themes, and a thematic analysis was completed on transcripts of workshops to identify key themes from participants’ experiences, recommendations, and discussions, using the 6-phase approach to thematic analysis as described by Braun and Clarke [38]. Reflexivity during content and thematic analyses was supported by regular meetings between researchers, which allowed consideration of themes and alternate explanations. Demographic and evaluation surveys were analyzed using descriptive statistics and content analysis, as described earlier.

### Co-Designed Resources

At the end of the study, co-designed versions of the resources were finalized, and a graphic designer was used to compile the final co-designed resources. An infographic was created to provide feedback to participants and distribute information about the study [39], and a website [40] was created to house and make publicly available the final co-designed resources and gather further feedback on them.

### Ethical Considerations

Ethical approval for this study was obtained from the Sydney Children’s Hospital Network Human Research Ethics Committee (HREC) (protocol 2023/ETH00995) and Charles Sturt University HREC (protocol H23804). Consent and child assent were obtained from all co-designers, including consent for publication.

## Results

### Child and Parent Co-Designer Demographics

Thirteen neurodiverse and developmentally diverse children (girls: n=6, 46.2% and boys: n=7, 53.8%, age range 5‐11, mean 8.6 y, SD 1.79 years) and 12 parents (mothers: n=9, 75% and fathers: n=3, 25%) from 9 families took part in the co-design study. Ten (83%) of the 12 parents answered a prestudy survey. All parents reported that they had never previously been given resources or advice on being active with their child. Further demographic information is listed in Table 3.

**Table 3.** Child and parent co-designer demographics.

Characteristic	Value, n
Parent age range (y)	
25‐29	1
30‐34	1
35‐39	4
40‐44	2
45‐49	2
Parent health issues limiting X-gen PA^a^^,^^b^	
None	9
Yes	1
Child health issues limiting X-gen PA^c^	
None	8
Yes	2
Additional issues identified by parents as limiting engagement in physical activity with their child^d^	4 responses

a X-gen PA: cross-generational physical activity.

b Open-text comment: “Chronic fatigue, limits my energy levels.”

c Open-text comments: “Just need to be careful as he has a connective tissue disorder which means his joints are unstable. No hard contact sports, just gentle activities” and “No energy to be active. Gets tired easily.”

d “ADHD: boredom, hyperfixation and transitioning hinders us both.” “Work/school schedule especially during winter.” “[name of child] is no fun to play with eg, listening, sticking to rules, needs to control everything.” and “I need to lose weight to feel like doing more.”

### What Were Families’ Experiences of Using the Prototype Resources?

All child and parent co-designers reported they were able to complete their planned session of X-gen PA. A parent said:

*I liked how it [the planning prototype] was all set out and how we ended up with a really good plan of what to do.*

Families noted the prototype planning resource helped them to organize X-gen PA and gave them a structure and language to debate what the episode would look like by considering all aspects of it. A father described how the prototype resource was used successfully as a reference to cease the frequent rule changes that their child tended to impose:

*It was so good, to have a reference, to be able to say "hang on a minute, this is what we decided.” Game-changing actually*.

A couple of families had to adjust their plans, and they reported that these adjustments were accommodated without difficulty. For example, a mother said:

*The planning tool helped us even when there was some adjustment in timing. There was no arguing, there was no ’yeah, but!*

There was consensus that the prototype resources were useful and user-friendly:

*It’s good, it‘s really easy for [child name] to understand and fill in. I found it pretty user friendly.* [Parent]

Families noted that the prototype resources included aspects of X-gen PA they had not considered, such as *physical and emotional safety* and *stopping* in the prototype planning resource. And a parent noted the following about the prototype *doing* resource:

*I thought this resource included things that I hadn' t consciously thought about. Like supporting child development for things like problem solving, social skills, communication... It puts a positive emphasis on trying to get out and do more activity together. Reading something like this reminds us why we should do it.*

Children said they “liked planning” and that the prototype resources were “good” as they gave them “different ideas of what to do.” The prototype *planning* resource encouraged co-participation in PA, as evidenced by one parent who observed that:

*if we hadn‘t planned this, I would have just sat down and watched them.*

There was consensus among families that using the prototype planning resource allowed children to know exactly what was happening. Using the resource reduced confusion and questions from children, which allowed all child and parent co-designers to have a better experience. For example, one mother said:

*I think it helped to stop a lot of the questioning that we get when we say we' re going to go and do something. We set it out, they know what they' re doing. … This allowed us to say it all at the beginning and then it allowed for a calmer results because they were part of the process.*

Children concurred, for example, one child said:

*It was really good because we had everything planned. I knew what we were doing.*

Some elements of X-gen PA covered in the prototype resources families had not considered planning before. For example, *how* to do the activity, *managing emotions,* and *stopping*. Families noted that planning improved their engagement and experience:

*We usually just get in and just do it. And not thinking about how and what the word ‘ how’ meant in that context. Normally if we go for a walk, it’s just come on let’s go for a walk but we haven' t really thought about it, we just do it. Thinking about how makes you think about what obstacles you do need to overcome to doing it.*

[Parent]

And a child said: “I like having rules because then we argue less.” Parents agreed, noting that planning enhanced their enjoyment by reducing arguments and allowing them to go “at a pace that works for them.”

In the second co-design workshop, some children also demonstrated an appreciation of how managing emotions could be used during other PA. For example, a child said:

*When I played handball at school, a boy lost and got really angry and bounced the ball really hard and hit his leg*. *He did not have a plan for keeping safe for big emotions*.

Likewise, child and parent co-designers commented that they found the *stopping* section useful but had not considered discussing or planning this aspect before. For example, a parent said:

*Talking about stopping beforehand is a great idea because we were able to plan what it would look like*.

The importance of planning this aspect was underlined by the wide variation between child and parent time expectations. For example, a child said: “I could spend the whole day doing this.” Parents noted how having an end point that was clear helped their children. One parent noted that it helped their child engage in the activity for longer periods, and another said:

*[Child name] is autistic and he can be rigid and things need to be in a certain order, and change does not come easily. So that stopping one ... is huge for him, cause you heard him, “3 overs each at 6 balls*.”

Parents also observed that knowing when PA will end helped them engage more fully with their children and the “confidence that it will end before everyone becomes tetchy and tired.” This allowed the activity to end on a positive note, without conflict.

### What Changes Were Made to the X-Gen PA Guidance Resources?

In total, 62 changes were made to the prototype guidance resources during the co-design process. Tables in MultimediaAppendices 3  4 detail the 35 changes suggested by child and parent co-designers and 27 changes made based on researcher co-designer observations. Textbox 1 provides a summary of the key changes from among these.

Textbox 1. **Summary of key changes made to resources based on co-designer contributions.**

1. Planning resource was changed to reflect the order most families addressed their planning in

For example, *Where* and W*hat* were combined into one section and moved up to after *Who*

2. Suggestions of activities were added to sections that did not have them

- *“*The bits of the resource which have lots of suggestions are awesome ... because it triggers ideas and helps us get more out of planning with him*”* (Parent).

3. Examples were added to sections to help understanding

- Some children found some of the ideas hard to grasp. For example, *Managing emotions,* they talked about being sad if they got hurt. Amended resource to provide examples of why emotions may become big.

4. Negotiation in planning needed additional support

- Several aspects in resource amended. For example, emphasized importance of hearing from both children and parents in *who* section. In *Reviewing together*, encouraged participants to work with their activity partner to help improve each other’s experience.

5. Support was required not just for planning the activity but considering the preparation too

- For example, packing swimming bag, remembering goggles.

6. Resource was amended to remind families of a simple definition of PA

- “I wouldn'’t have thought that just handball was physical activity because to me physical activity is walking, running, swimming, anything that they label as fitness. So that was even an eye-opener, oh this is quite fun, and it’s easy to do, and it’s still being active!” (Parent)

7. Resource was amended to remind families that PA didn’t need to take a long time

- Parents reported that using this resource helped them appreciate that being active with their child need not take a long time after some commented that the time to engage was more achievable than they had realized.

8. Simplified some language that children identified as hard

- “I think you can improve it by getting rid of some of the big words so it’s easier for other children to read.” (Child)
- For example, changed *routine* to *everyday*

9. Added *Hard Words* section

- Children showed very variable ability to read and understand some words but resources are for families to read together. Retained some *hard words*, eg, *agility, self-esteem,* they were highlighted in orange in the resource and an explanation provided in the *hard words* section at the end of the resource to support parents to explain them

10. Parents advised that they would like to normalize that no-one wins all the time

- Added to *Some families compete and measure against each other*
- I think it’s really important just to remind them that “It’s okay if you don’t win, as long as you’re better at—getting better within yourself.”

### Has Using the Resources Changed the Way That You and Your Parent or Child Are Active Together?

Five children and 3 parents (n=8) answered a post–co-design survey. Six of these co-designers (children: n=3, 37.5%, parents: n=3, 37.5%) answered *yes* to the abovementioned question. One child answered *no*, and one child answered *don’t know*. Child and parent co-designers were invited to tell us more. Table 4 provides the themes and related examples arising in responses from child and parent co-designers.

**Table 4.** Has using the resources changed how you are active together? Themes and examples based on responses from child and parent co-designers.

Themes	Child responses	Parent responses
Shared themes	*Do it more together (n=3)* *We do more with them* (n=3).	*Do it more together* (n=1) *I try to be more involved and play with them instead of just sending them to play on their own*
Child and parent only themes	*Plan PA more* (n=1) *We talk about it more. I know what we are going to do*.	*Greater insight into child’s interests* (n=1) *It helped me see that what I consider a fun activity is not what [name of child] likes to do. That didn’t occur to me*. *Increased child agency* (n=1) *The kids feel like they have more agency over the activities we do together when we plan, rather than me picking the activity and us doing that*.

### Was the Co-Design Approach Authentic?

#### Feedback From Advisory Groups During Co-Design

Child advisors during the second and third advisory group meetings were predominantly positive about the changes made to the prototype resources in response to child feedback. However, some changes attracted more approval than others, and scores given by child advisors ranged from 5 (a big smiley face) to 2 (a slight frown). For example, child advisors rated the addition of a suggestion that children and parents swap roles, and the child takes on the role of teacher or coach, in the *Being active together supports child development* section of the *Doing Physical Activity Together* prototype resource, with big smiley faces. A child advisor wrote: “This is because I understand this and it makes me want to say this to my parents.”

They reported less enthusiasm for other changes. For example, making more space for writing in the blank plan by making it 2 pages instead of 1 was rated neutral to smiley face (3-4) by child advisors. A child advisor wrote: “It‘s not a big change but it is still a good change.” Changes were made to simplify language and provide a small dictionary of *hard words* in response to child and parent feedback. One child advisor did not like the changes made to simplify the language that *they* understood. They scored this change with a slight frown (2), noting:

*Because some of the words like “bonding” I know about and to me it‘s unfair that I know a word and you take it out.*

Likewise, support for changes made to the prototype resources was received from the adult advisory group members during the research. While adult advisers reported they appreciated this transparent approach, no additional advice or commentary was received from them.

#### Feedback Post Co-Design Process

**Figure 2.**
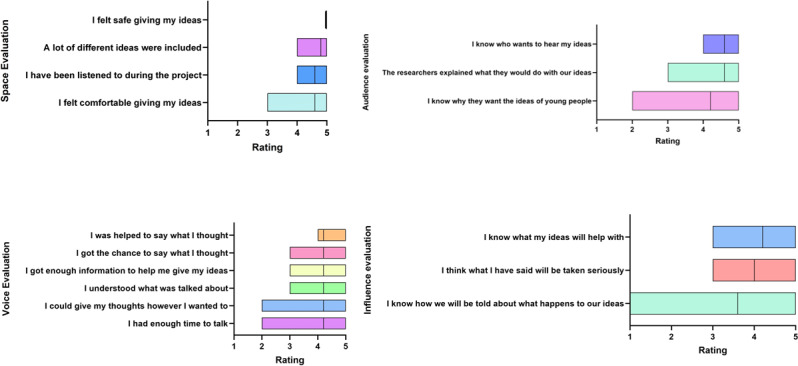
Child co-designer evaluation of co-design process (mean scores and range).

All children who participated in the third co-design workshop answered the co-design evaluation survey (n=5), providing a score of 1 to 5 stars for each of 16 statements. Figure 2 shows the findings from the survey, in which questions were based on Lundy’s framework of participation [40]. The statement, *I felt safe giving my ideas*, was scored 5 stars by all child co-designers. Only one statement received an average score lower than 4 stars. The statement, *I know how we will be told about what happens to our ideas*, scored an average of 3.6 stars (range 1‐5).

The bars in the figure depict the lower and higher limits of the range of scores, and the line that dissects each bar indicates the mean of the scores.

Questionnaire adapted from the Government of Ireland Participation Framework for Children and Young People Participation in Decision Making (Government of Ireland, 2021).

Three of 12 (25%) parent co-designers took part in the co-design evaluation at the end of the project (Textbox 2).

Three of 5 eligible adult advisory group members (60%) completed the questionnaire provided to them as part of the evaluation of the co-design process (Textbox 3).

Textbox 2. **Parent co-designer evaluation of co-design process (n=3).**

1. All parent co-designers ranked the following statements on a 5-point Likert scale: 5=strongly agree

- I was given enough information to allow me to form a view about co-design
- I was given the experiences needed to form a view about co-design
- I was given enough time to express myself during co-design
- I felt comfortable giving my opinions about co-design
- I have been listened to by the researchers during the project
- I know what will happen to my opinions

2. What worked well in your co-design experience?

Two themes were found

- Co-design experience provided opportunities to share their experiences and opinions. One parent co-designer said, “I felt the whole process was valuable and gave me the ability to share my experiences and opinions. The resources worked well and it gave me some great ideas to bring to home life*.*”
- Two parent co-designers identified that the context and approach to co-design worked well. For example, a parent co-designer said, “Two way conversations and easy explanations.*”*

3. What could be improved from your co-design experience?

Nothing (n=3)

Textbox 3. **Adult advisory group evaluation of co-design process (n=3).**

1. Did the study provide opportunities for children and parents to participate in co-design?

100% (n=3) replied Yes

Two themes identified invitation to comment on the answer above

- Engagement with children and parents (n=2)

-  “Children involved in the advisory group were treated with respect and engaged with in a friendly manner. They appeared very comfortable to give their opinions and ideas.”

- Multiple rounds of co-design (n=1)

-  “There were several rounds of co-design sessions. This enables the refinement of themes from the sessions.”

2. How much influence were the children and parents able to exert upon the co-design of the resources?

5-point Likert scale (1=*not much influence*, 5=*a lot of influence*).

33% (n=1) scored 4

66% (n=2) scored 5

3. What impact do you think the co-designed approach had upon the PA resources?

User-friendly end product (n=2)

Tailored to the study population (n=1).

4. What worked well with the co-designed approach? (Two respondents provided more than one answer).

Structured, user-friendly approach (n=1)

Time to allow children to fully engage (n=1)

Child advisory group well supported by child voice facilitator and advocate (n=1)

Transparent approach (n=1)

Communication between adult and child advisory groups (n=1)

5. What could be improved from your co-design experience?

Nothing (n=3)

#### Research Team Reflections Upon Co-Design Approach

The research team identified 2 elements of the co-design methodology that may have impacted child engagement in the study: the amount of information provided to child advisors and challenges in communicating with child advisors outside face-to-face sessions.

First, the purpose of doing the research and the importance of hearing from children were discussed with child advisors during the first child advisory group meeting but omitted from the second meeting of the child advisory group. This resulted in a child advisor voicing uncertainty to the CVFA during the second meeting about why adults wanted to hear their thoughts on the changes that were being made to the resources. Upon reflection, the researchers realized that while we included a brief recap of terms of reference at the beginning of each adult advisory group meeting, we had neglected to support child advisors in this way at the second child advisory group meeting. Thus, at the beginning of the third meeting of the child advisory group, a researcher was invited to join the group and facilitate a further discussion with child advisors about why their views were important.

Second, child advisors were sent an email update from the CVFA after each adult advisory group meeting. The intent of this communication was to close the communication loop and ensure that child advisors did not experience the frustration children have previously highlighted, that they are not informed about research progress and outcomes [27]. The email update was sent to each child’s parent’s email address to inform the child advisors of what the adult advisory group wanted to feed back to the child advisory group. Communicating with primary school children via their parents’ email addresses was considered an important ethical approach, which ensured that parents were aware of and had control over interactions between the researchers and their children. However, it was found at the second child advisory group meeting that not all parents had shown their children this email or child advisors could not remember seeing or hearing about it. To address this issue, the feedback provided in the emails was reviewed with the child advisors at the beginning of the second and third child advisory group meetings. However, this finding does identify that the researchers had no way of knowing whether child co-designers were shown other emailed communication, such as the infographic sent to all families, via parents’ email addresses, at the end of the project.

## Discussion

### Principal Findings

This exploratory co-design study identified ways to increase usability and acceptability of guidance resources intended to increase family knowledge of how to plan and engage in PA together and evaluated the co-design approach it used. An asynchronous approach to co-design was taken to allow the development of theory-informed prototypes for families to trial and critique [41]. Key changes recommended by children, parents, and researchers to optimize the resources included changing the ordering of the planning resource to reflect the way that most families used it, adding a *hard words* section to aid readability, support to normalize that no-one wins all the time, additional examples of the types of PA families could do together, and assistance for negotiation among families to ensure that the planned PA was mutually desirable.

The secondary aim of the study was to evaluate the co-design approach. Overall, child and adult evaluators perceived the co-design approach to be appropriate and responsive and identified several elements of the co-design methodology that facilitated child and parent voices and increased methodological rigor. For example, the use of a CVFA to facilitate child advisory groups enabled safe spaces for children to contribute and evaluate changes made to the resources while ensuring independence and transparency of this aspect of the evaluation. Findings from the child evaluation and researcher observations resulted in 2 suggestions for co-design researchers to consider when conducting co-design research with children, which are discussed further later in this section.

Previous research has found that parents are already convinced about the physical health benefits of regular PA for their child but that has not changed their behavior in supporting their child’s PA [42], and PA participation rates in children are still low worldwide [6]. It is also likely that some parents, who are not regularly active themselves, may not have the PA vocabulary necessary to enable, and undertake, successful episodes of PA with their children. Parent co-designers in this study discussed how the resources encouraged them to consider aspects of doing PA with their child that they had not considered before, such as how engaging in PA together supported their child’s emotional and social development. It is hypothesized that the final co-designed resources [40] may provide families with greater insight into the wide range of health and well-being benefits of being active together. The resources were also written for both PA partners (children and their parents) to use together, as it is further hypothesized that planning and talking about PA together may influence family PA behavior and support for PA within families. However, both hypotheses will require further investigation with larger numbers of participants.

None of the participating families had previously received resources or advice on parents being active together with their neurodiverse and developmentally diverse children. The small sample size of this study means that this finding should be interpreted with caution, but it nonetheless is concerning because PA is an important intervention for these child populations [10]. Social PA interventions, such as X-gen PA, not only provide opportunities to practice physical skills and gain physical health benefits but also offer opportunities to practice prosocial skills, such as communication skills, taking turns, and self-regulation [10,43]. Provision of information to support families with neurodiverse and developmentally diverse children to take part in positive X-gen PA experiences is required. Development of paper-based or online resources, such as those created in this study, provides one possible way to deliver this support. Some families suggested that accessing the information via an interactive app would be fun, but it is acknowledged that the development cost and subsequent subscription costs of such an app might limit equitable access by excluding lower-income families [44].

The findings of this study suggest that the planning resource may help families consider together what obstacles they need to overcome to be active together. Importantly, parents reported they enjoyed their subsequent planned episodes of X-gen PA, indicating their children asked fewer questions and were more receptive to stopping PA at the end of an episode as planned, allowing for calmer experiences of X-gen PA and greater confidence that episodes would end without conflict. Basic psychological needs theory [45] may provide further explanatory insights into these experiences of success. The resources addressed all 3 needs that underpin the sustainment of intrinsic motivation for a behavior—relatedness, autonomy, and competence [45]. For example, planning stopping meant that children and parents gained insights into the often substantial differences between child and parent time expectations, resulting in conversations to plan mutually agreed-upon time frames for X-gen PA. Stopping of episodes of X-gen PA has previously been noted in the literature as an aspect of X-gen PA that children who were neurotypical found frustrating due to greater parental control over the cessation of a PA episode [18]. Creating a structured environment for children with neurodiversity, such as planning PA, has been identified as a key strategy to help children with neurodiversity manage their behavior [46]. Planning stopping also gave children with neurodiversity knowledge and preparation time to transition from one activity to another, which can help children with neurodiversity manage their emotions [46].

Previous parenting research has found that planning PA opportunities was identified as a key self-regulation strategy that parents who identify as an active parent perform for their family [47]. Research suggests that both role and social identities play a part in maintaining health-enhancing behaviors such as PA [44,48]. Role identity research has found a positive link between PA frequency and having a strong exercise identity [49]. Social identity theory acknowledges that people identify themselves by both their personal identity *and* a wide variety of social identities [49]. Thus, it is hypothesized that encouraging and supporting children to have both a voice and influence over the child-parent PA partnership may positively influence both a child’s identity as an active person and foster a shared “active family” identity more than resources directed solely toward parents and carers, in which children are assumed to be the passive recipients of parental decision-making.

Two recommendations for family co-design have emerged from the process evaluation of our exploratory co-design study. First, it is important to remind child advisors at the beginning of each child advisory group meeting why children’s voices are important to hear [27,28] and of the purpose behind the child advisory group (eg, terms of reference). We note that establishing agreed terms of reference has been identified in recent research on co-design studies as one factor that may enhance co-designer cooperation and understanding of their roles as co-designers [50]. Second, this study found that communicating to children via their parents’ email addresses did not ensure that child advisors were provided with information about research progress and thus we are unable to determine whether child co-designers saw the infographic depicting the research outcomes that was written for families [39]. The HREC approved the researchers to communicate with the child co-designers via their parents’ email addresses, and we acknowledge the importance of ensuring children’s safety and privacy. However, not closing the communication loop with children has been identified in previous research as a source of frustration by children [27]. Our study suggests there may still be a role in sending out research summaries by mail, perhaps addressed to the whole family, to increase the likelihood of children accessing research outcomes. Previous research has found that children enjoy receiving research outcomes in the mail and suggest sending research in multiple formats, such as summaries, coloring books, and posters [51].

### Limitations

The small number of co-designers and advisory group members in this study was recruited from a single rural Australian LHD, using a purposive sampling approach. We acknowledge that readers will need to determine the transferability of our findings to other populations; thus, we have provided detailed methodology, including descriptions of settings and participants. We hope our transparent approach will allow readers to consider the relevance of the study’s findings to other populations and contexts.

Fewer child and parent co-designers took part in the second phase than in the first phase of the study. A gap of two and a half months (school term) between phases is likely to have contributed to this reduction in numbers. In future research, we recommend, where possible, recruiting more child and parent co-designers to reduce the impact of attrition [51] and completing studies without such long time gaps to maintain momentum and interest. However, it should be noted that we used holiday periods because parents informed us in this study that engagement was more likely during holidays, as term-time schedules are too busy. As this study was conducted in Australia (southern hemisphere), it was not possible to conduct the study in one (summer) holiday period due to the impact of Christmas during this time, but conducting the study over a continuous 6-week period would have been ideal. It should be noted that the reduction in engagement in the second phase of the study subsequently impacted the number of children and parents who provided an evaluation of the co-design approach at the end of the study, as child and parent co-designers were invited to complete paper-based evaluations at the end of the second phase workshop. Future studies could also consider a continuous evaluation approach through the use of a short survey at the end of each workshop. Finally, the possibility of desirability bias should be noted. Steps were taken to address this risk by empowering co-designers and advisory group members, such as by employing a nonresearcher (the CVFA), doing activities that promote collaboration to disrupt power hierarchies (eg, playing games and anonymous feedback) [52], and use of multiple methods to provide different ways for co-designers to collaborate and express themselves.

The final co-designed resources are freely available under a Creative Commons License [40]. Further research is needed to test the usefulness of the co-designed X-gen resources in a broader rural population. It is suggested that such research could examine the impact of the resources on family PA literacy, family cohesion, and PA identity, and their use during holidays rather than their impact on an individual’s weekly PA total.

### Conclusions

In this study, child and parent co-designers provided multiple suggestions to improve the usability and acceptability of guidance resources that had been compiled by experienced physiotherapists, underlining the importance of consumer involvement in resource design. The co-design methodology supported discussion and planning of X-gen PA among rural families with neurodiverse and developmentally diverse children. Future research is needed to evaluate whether these resources can positively impact family PA behavior in the longer term and across broad populations. Evaluating co-design approaches is an important way to provide transparency of the processes involved and to protect consumers against tokenistic co-design approaches. This study highlighted the need for consideration of how to convey study findings to child co-designers.

## Supplementary material

10.2196/92658 Multimedia Appendix 1 Elements of co-design approach that facilitated family voices evaluated using Lundy's voice model checklist for participation.

10.2196/92658 Multimedia Appendix 2 Checklist for sufficiency of reporting of co-design approach.

10.2196/92658 Multimedia Appendix 3 Child and parent co-designers' suggestions and researcher co-designers’ observations on Planning Physical Activity Together resource.

10.2196/92658 Multimedia Appendix 4 Child and parent co-designers’ suggestions and researchers’ observations on Doing Physical Activity Together resource.

## Abbreviations

CVFA: child voice facilitator and advocate
HREC: Human Research Ethics Committee
LHD: Local Health District
PA: physical activity
X-gen PA: cross-generational physical activity
