# Supplementary material for: Physical Activity Guidance Resources for Rural Families of Neurodiverse or Developmentally Diverse Children: Exploratory Co-Design Study
Source: JMIR Pediatr Parent. 2026 Jul 14;9:e92658. doi: 10.2196/92658 (PMC13367945; doi:10.2196/92658)
Supplement: Multimedia Appendix 1 [file pediatrics-v9-e92658-s001.pdf]

Appendix 1. Elements of Co-design Approach that Facilitated Family Voices Evaluated Using Lundy's Voice Model Checklist for Participation (Ireland Department of Children and Youth Affairs, 2015).

SPACE	Provided a safe and inclusive space for co-designers to express their views
**Were co-designers' views actively sought?**	• Yes. All families, who agreed to be contacted about the study, were invited to join the study
**Was there a safe space in which co-designers can express themselves freely?**	• Children and parents worked together for first two workshops<br>• Fun, friendly, accepting and respectful approach by researchers and CVFA<br>• CVFA ran the child advisory group to enable a safe space for children to critique the project<br>• Workshops and child advisory groups took place in local library which was a familiar space to families<br>• Workshops and child advisory groups took place in a quiet, private room, with minimum distractions<br>• Workshops and child advisory groups took place during school holidays, as identified by parents as a suitable time
**Were steps taken to ensure that all co-designers could take part?**	• Recruited populations that have been seldom heard<br>• Child and Adult Advisory Groups were established and all co-designers were invited to join their respective group
**VOICE**	Provided appropriate information and facilitate the expression of co-designer's views
**Were co-designers given the information they need to form a view?**	• The project was designed so that co-designers' trialed the resources before being asked to provide their views
**Did co-designers know that they did not need to take part?**	• The information sheets clearly explained that they did not need to take part, and could stop at any time<br>• Researchers actively followed cues from family, on literacy levels, development and engagement
**Were co-designers given a range of options as to how they might choose to express themselves?**	• A range of activities were used to maximize participation opportunities for everyone<br>• Co-designers were given choices on how to engage

**AUDIENCE**	Ensured that co-designers' views were communicated to someone with the responsibility to listen
**Was there a process for communicating co-designers' views?**	• Yes, researchers, who were experienced in working with children, conducted the co-design workshops<br>• Yes, the CVFA acted as an advocate for the child's voice in the adult advisory groups and related a response back to the child advisory group
**Did co-designers know who their views were being communicated to?**	• Yes, introductions were made at the beginning of every workshop and advisory group
**Did that person/body have the power to make decisions?**	• Yes, the researchers who conducted the co-design workshops were responsible for analysis and making changes to prototype
**INFLUENCE**	Ensured that co-designers' views were taken seriously and acted upon, where appropriate
**Were the co-designers' views considered by those with the power to effect change?**	• Yes
**Are there procedures in place that ensure that co-designers' views have been taken seriously?**	• Separate child and adult advisory groups were set up to advise on the co-design approach<br>• The CVFA was responsible for ensuring that child advisory group voices were heard by adult advisors<br>• All co-designer suggestions were collated and considered, and researcher consensus reached on further action. Child advisory group consulted on some co-designer suggestions and outcomes that pertained to child feedback. Adult advisory group consulted on all co-designer suggestions and researcher observations and outcome of researchers' decisions
**Have the co-designers been provided with feedback explaining the reasons for decisions taken?**	• Members of the advisory group received feedback explaining the reasons for decisions taken<br>• Findings from the project were compiled into an infographic that was shared with all co-designer families. The infographic had a link to the three co-designed resources developed from the project

Notes: CVFA: child voice facilitator and advocate. In the table above *children* has been replaced with *co-designers* to encompass both child and parent co-designers.
