# Supplementary material for: Physical Activity Guidance Resources for Rural Families of Neurodiverse or Developmentally Diverse Children: Exploratory Co-Design Study
Source: JMIR Pediatr Parent. 2026 Jul 14;9:e92658. doi: 10.2196/92658 (PMC13367945; doi:10.2196/92658)
Supplement: Multimedia Appendix 2 [file pediatrics-v9-e92658-s002.pdf]

Appendix 2. Checklist for sufficiency of reporting of codesign approach.

CHECKLIST ITEM	LOCATION OF ITEM (under heading) IN PAPER
**1. Is the intention behind the participatory approach described?**	Introduction
**2. Is there a description of where the participatory approach took place?**	Setting
**3. Is there a description of who facilitated the participatory approach?**	Researchers, Partner Organisations, and Child Voice Facilitator and Advocate
**4. Is there a description of who was involved in the participatory approach?**	Family Co-designers and Advisory Groups
**5. Is a description of the intervention or resource provided?**	Development of evidence-informed prototypes resources
**6. Are descriptions of the phases and methods of participatory approach provided?**	Tables 1 and 2
**7. Are descriptions of the intensity and schedule provided?**	Tables 1 and 2
**8. Is the intention of participatory approach evaluated?**	Yes
**9. Is there a description of how shared decision making was facilitated?**	Yes, Advisory Groups describes how advisory groups were invited to review suggestions and changes to resources and comment
**10. Do the authors report on end user input into the final resource or intervention?**	Tables 4, S3 and S4

Freire et al (2022)
