# Supplementary material for: Physical Activity Guidance Resources for Rural Families of Neurodiverse or Developmentally Diverse Children: Exploratory Co-Design Study
Source: JMIR Pediatr Parent. 2026 Jul 14;9:e92658. doi: 10.2196/92658 (PMC13367945; doi:10.2196/92658)
Supplement: Multimedia Appendix 3 [file pediatrics-v9-e92658-s003.pdf]

Appendix 3. Child and parent co-designers' suggestions and researcher co-designer' observations on *Planning Physical Activity Together* resource.

General observations and comments		
**Child and parent** co-designers' comments and suggestions	Interpretation and action	Changes to resource
• "Putting it on an app would be good. It would be easier and more fun to use. And then we could refer back to it. Pieces of paper get lost". (Parent)	Suggestion acknowledged. Would require funding.	0
• "It would have been good to have had access to these resources earlier. I feel like, if you're trying to get the kids input, about 5 or 6 [years] would be good. (Parent)	Suggestion acknowledged. Intention is to make available to families with children of primary school age.	0
• "The bits of the resource which have lots of suggestions are awesome … because it triggers ideas and helps us get more out of planning with him". (Parent)	Reviewed sections of resource that do not have suggestions, suggestions added to *why* section.	1
Researchers' observations	Action	Changes to resource
• Most families address *what* and *where* at the same time.	Amended resource to have *what* and *where* in one section.	1
• Wide variation in how families used resource - some reading verbatim, some reading most sections but filling in other sections, such as *when* without referring to resource.	Nil action.	0
• Negotiation in some families needed further support. Some parents made it more about what the child wanted to do rather than coming up with something they both wanted to do.	Amended resource to emphasize importance of hearing from both children and parents in *who* section. Added warning that end of plan there are questions about whether they made the plan together and if they are both happy to try their plan.	3
**Summary planning sheet**		
**Child and parent** co-designers' comments and suggestions	Interpretation and action	Changes to resource
• "There could be a bit more space for writing" (Child)	Summary resource expanded from 1 to 2 pages to allow more space for children to fill in it.	1
Researchers' observations	Action	Changes to resource
• *Who* and *summary of (name) and (name)* confused children. Essentially same thing.	Removed *summary of (name) and (name)*.	1
• Observed that preparation for activity might be an issue. E.g. packing swimming bag, remembering goggles.	Amended resource to include prompt about planning.	1

**Who**		
**Child and parent** co-designers’ comments and suggestions	Interpretation and action	Changes to resource
• Mother suggested prompting families to consider other family members who might not be taking part. Do they need prompting to make plans for another sibling or family member if they are not joining in the activity.	Amended resource to add prompt to consider if alternate plans need to be made for family members who are not taking part in the activity.	1
Researchers’ observations	Action	Changes to resource
• Child had difficulty identifying who was in their family.	Added *carer* to first section to broaden definition of family.	1
**What**		
**Child and parent** co-designers’ comments and suggestions	Interpretation and action	Changes to resource
• “I wouldn't have thought that just handball was physical activity because to me physical activity is walking, running, swimming, anything that they label as fitness. So that was even an eye-opener, oh this is quite fun, and it's easy to do, and it's still being active!” Parent	Amended resource to contain simple definition of PA	1
Researchers’ observations	Action	No of changes to resource
• Some families used the list of activities rather than coming up with their own.	Amended resource to emphasise that the list is not exhaustive	1
• Some activities required more detail. E.g. rather than just *swimming.* Clarify if this means laps or is this *play* in the swimming pool.	Amended *how* section in resource to suggest further discussion of the activity may need to happen.	1
• Putting *how* straight after *where* and *what* would support clarifying activity.	Amended order of resource.	1
**Why**		
No suggestions from **child and parent** co-designers in this section.		
Researchers’ observations	Action	Changes to resource
• Most families asked why they are doing the activity, rather than why they are doing the activity together.	Amended resource to provide examples that include reasons for doing the activity together.	1

When		
No suggestions from **child and parent** co-designers in this section.		
Researchers' observations	Action	Changes to resource
• Families tended to address this section once they had sorted out *what* and *where*.	Amended order of resource.	1
• Useful to add in considerations about different people's schedules. Children were observed to give repeated suggestions that did not fit in with their parents' schedules. This would mean children are not suggesting times and days and parents always saying no.	Suggestion added to resource, that families might want to use a calendar when discussing this aspect, so that everyone can see times that people are busy and times when their activity partners may be available.	1
• Parents noted that using this resource helped them appreciate that being active with their child need not take "a long period of time, half an hour, 20 minutes is fine". They felt this was more achievable than they had realised.	Added reminder in resource that doing physical activity together needn't take a long time.	1

Where		
No suggestions from **child and parent** co-designers in this section.		
Researchers' observations	Action	Changes to resource
• *Where* was discussed with most families as part of *what.*	Amended resource. Already addressed in *what* section.	0

How		
No suggestions from **child and parent** co-designers in this section.		
Researchers' observations	Action	Changes to resource
• Some children became distracted with measuring height/arm length differences rather than figuring out if there were any other differences.	Amended resource to suggest family figure out what their differences are, before providing examples.	1
• Some families did not appear to have insight into why this section was important.	Amended wording to emphasise need to "agree with your partner" and how "coming up with a clear plan together helps everyone prepare and manage their feelings during an activity".	1
• A prompt about gathering together equipment for activity would be useful.	Amended resource. Section at end of how. Is there anything you need to prepare for the activity. Such as pack a swimming bag.	1

Keeping safe		
No suggestions from **child and parent** co-designers in this section.		
Researchers' observations	Action	Changes to resource
• Some families already had a plan to follow to manage big emotions.	Amended resource to suggest to families that if they already have a plan that is working for them, they should use this plan during activity together.	1
• Some children found the idea of managing emotions hard to grasp. They kept referring to if someone got hurt and how they would manage being sad.	Amend resource to provide further examples of why emotions may become big.	1
**Stopping**		
No suggestions from **child and parent** co-designers in this section.<br>No observations were made that resulted in changing the resource.		
**Reviewing**		
**Child and parent** co-designers' comments and suggestions	Interpretation and action	Changes to resource
Some families didn't like the open ended questions. However, other families supported open ended questions as they were keen to encourage their children to talk about their feelings. Families suggested smiley faces for younger children. "but I can see if the younger children were to do it by themselves pictures would be easy" (Parent).	Amended resource, see above. Also added, should we do this activity again	1
Researchers' observations	Action	Changes to resource
• Some parents were not comfortable identifying what they did not like.	Amended resource to remove question about like and dislike and added star rating and suggestions that they could tell their partner why they gave this score.	3
• Encourage participants to work with their activity partner to help improve each other's experience.	Amended resource to ask *how you can work together to achieve what each of you want?*	1
**Total number of changes to resource = 28**<br>**Number of child and parent co-designers' suggestions that led to changes = 5**		
