# Supplementary material for: Physical Activity Guidance Resources for Rural Families of Neurodiverse or Developmentally Diverse Children: Exploratory Co-Design Study
Source: JMIR Pediatr Parent. 2026 Jul 14;9:e92658. doi: 10.2196/92658 (PMC13367945; doi:10.2196/92658)
Supplement: Multimedia Appendix 4 [file pediatrics-v9-e92658-s004.pdf]

Appendix 4. Child and parent co-designers' suggestions and researchers' observations on *Doing Physical Activity Together* resource.

General comments		
**Child and parent** co-designers' comments and suggestions	Interpretation and Action	Changes to resource
• Parent suggested booklet form to make easier to read. Observation from researchers: some families missed pages when reading through prototypes.	Changed both resources to booklet form.	1
• "Perhaps you can put together a slogan, like a slip, slop, slap slogan to go with it to have that reinforcement" Parent.	Researchers thought resources cover too much information to be easily reduced to a slogan.	0
**Building and maintaining your relationship**		
**Child and parent** co-designers' comments and suggestions	Interpretation and Action	Changes to resource
• *Parent:* That's pretty much the only thing I thought about that was having less distractions. They feel like it's a good time to talk with us. I find they talk a lot more about how things are going with them in those activities.<br>*Interviewer:* The connection not just with you, as the parent, but<br><br>*Parent:* But with them [between siblings] as well.	1. Reviewed resource. Changed *interruptions* to *distractions* as research team agreed with parents that it was a more encompassing word.<br>2. Parents mentioned about connecting to siblings during PA too – resource talks about family relationships. No changes made as resource focused on X-gen not sibling relationships.	1
• "I think you can improve it by getting rid of some of the big words so it's easier for other children to read". (Child)	Variable understanding of *bonding* and *connecting* amongst children. Simplified wording in section. Replaced *bonding* and *connecting* with *sharing* and *relating*. Changed *routine* to *everyday*. Omitted word *'aspects'* from resource. Changed *opportunity* to *chance*.	5
Researchers' observations	Action	Changes to resource
• Observation - 'for example, by doing something healthy with them …' doesn't seem to link with the one before and it is a long and confusing sentence.	Restructured sentence.	1

Being active together supports child development

**Child and parent** co-researchers’ comments and suggestions	Interpretation and Action	Changes to resource
• *Parent:* I think the mental health side of it is really important to talk about with kids, so that they have a good understanding of how it’s positive. Like, “This helps your body produce those unseen endorphins,” I suppose. I think that's really important that we should talk to kids about their mental health from an early age, so they have a good understanding that “Hey, if you go outside, this is gonna make you feel better inside yourself.”	No changes added Discussed in *It’s a healthy way to spend time together.*	0
• *Interviewer:* Those ones are tricky ones, aren't they? Sport and exercise helps our body by training agility, strength, and reaction times. Do you know what agility means?<br>*Child:* I know what reaction it means.<br>*Interviewer:* What does that mean?<br>*Child:* Something happens like I get an allergic reaction to grass.<br>*Interviewer:* Ah, yeah. That's a good way to think about it. Reaction times in sport and exercise is that if—<br>*Child:* Is that like when you move?<br>*Interviewer:* Yeah, so if a ball's coming to you, how quickly you're able to go, "Oh there it is," and catch it.	Added *hard words* section to provide explanation of these words and some others.	2
• *Child:* These skills are useful for many other areas of life in addition to physical activity.<br>*Interviewer:* Thinking about maths again, are you? … Problem-solving and addition.	Explanation of *problem-solving* added to hard words section. Removed *in addition* from last sentence.	2
Researchers’ observations	Action	Changes to resource
• *Interviewer:* Hearing you talk about that, flipping that on its head could also be, “What do you find difficult about the sport that they enjoy, and can the children coach you on that part?”… Those problem-solving skills, communication, concentration, can also be done in reverse. … How can we meet in the middle?”<br>*Parent:* I think they would really enjoy that. I do. I've never thought about it like that, so that's a really good tip.	Suggestion added to *You might like to try* section. *Parent: can you think of an activity your child does that you find hard or don’t know much about? Could you ask your child to teach and help you to find some fun ways to practice it with them? This may provide you with opportunities to model learning from mistakes. Children often enjoy the chance to be the teacher.*	1

Practising sporting skills		
**Child and parent** co-designers' comments and suggestions	Interpretation and Action	Changes to resource
• "During the time difference between the two soccer games, (name of father) or I will play soccer with her, kick the ball, and stay active. Because that's not a told activity, she's quite happy to do something because we're not saying, "We're gonna go to the park," or "We're gonna go for a walk." It's something that happens because you're there, you might as well be doing something".	Opportunistic, informal practice worked well for this family. Added a section *parents tell us* which includes that not all children enjoy practising and that finding informal, opportunistic times with them may help.	1
• "He doesn't tolerate her not accepting doing stuff, I suppose. If she can't kick the ball on three feet—kick it three times, then she'll go, "Ah, sorry. I can't do that." He wants her to persist with that. He gets cranky that she won't persist. Then she gets cranky that he's cranky, and then it's not fun anymore".	In section *parents tell us* acknowledged that practising can be a frustrating time for all, and suggest they make a plan for managing big emotions, referring to planning prototype.	1
• "We cannot get her to practice. Even soccer in the backyard, she won't practice soccer in the backyard. She'll kick the ball with us in between soccer games and practice because we're not practising, right? She will not—even at home, I'm like, "Kick the soccer ball against the wall and practice stopping it." "No, I'm not doing that".	This parent is expecting their child to practice by themselves. Section added in *parents tell us* to emphasise that some children may find practise with them more enjoyable.	1
• Parent described how reluctant her child was to practice and was asking for more information on how much people needed to practice to gain skills.	Added section *Research tells us* with two messages. Practice takes repetition and that children may need help in identifying how they are improving.	2
• *Child: "*Encouraging".<br>*Interviewer:* "Encouraging. You think encouraging should be in there somewhere? … Do you enjoy the encouraging aspect of it?"<br>*Multiple Children: "*Yeah".	Changed *supporting* for *encouraging*	1

Some families compete and measure against each other

**Child and parent** co-designers' comments and suggestions	Interpretation and Action	Changes to resource
• "No real competition. No, everything's a competition for [name of child]. Even when we were doing our dance thing, my skills were being critiqued the whole time *[laughter]*. It's like, "Hang on. You've had dance lessons. I've never had dance lessons".	This parent is describing a child comparing their dancing skills to their parent. This aspect of comparing themselves to family members is covered in the resource lower down the page.<br>Added *competing together is about playing and learning to compete* to emphasise role that family may play in learning about competition.	1
• *Parent:* I think it's really important just to remind them that "It's okay if you don't win, as long as you're better at—getting better within yourself." I think I'm—even though I am fairly competitive, I like them to understand that it's about them getting better themselves, personally, more so than winning every time.<br>*Interviewer:* I guess, as you said, that's that positive reinforcement of the effort and the—<br>*Parent:* I think that's really important. Yeah.<br>*Interviewer:* self-achievement. Potentially, it sounds like a bit of modelling of losing as well.<br>*Parent:* That's right. Yeah. I think it's really important to do all of that. I think everything in there is really good.	Simplified some of the language.<br>Added another section to emphasise that no-one wins all the time.	2

Parents use it to support and care for their children

**Child and parent** co-designers' comments and suggestions	Interpretation and Action	Changes to resource
• Facilitate - child did not understand word.	Changed to 'help'.	1
Researchers' observations	Action	Changes to resource
• Observation – children's delight in receiving their PA toys for taking part in research.	Added a section: Research tells us addressing the importance of providing children with equipment and toys that help children to be active, such as balls, bats and bikes.	1

It’s a healthy way to spend time together		
**Child and parent** co-designers’ comments and suggestions	Interpretation and Action	Changes to resource
• *Parent: T*he risk of developing health conditions is too difficult.<br>*Interviewer:* What can we say instead? Reduced chance of?<br>*Parent:* Of getting sick?<br>*Parent:* Cause they’re reading heart, lung conditions, type 2, and cancer there. [name of child] would be like, “I don’t know what that is.”	Amended resource to *reduce your chance of getting sick*.<br>Still included list of health conditions because researchers think those words are understood by parents and important information for parents.	1
• *Parent:* Weight, it could be negative.<br>I suppose it is quite negative.	Amended resource to remove reference to weight. Replaced with *keeps you moving and uses up extra energy*.	1
• *Parent:* Reduce anxiety and depression is too much<br>*Interviewer:* Do we say worry and low mood or something like that?<br>*Parent:* Yeah	Changed to *makes you less worried and gloomy.*	1
• *Interviewer:* Is there anything else you’ve talked to your kids about?<br>*Parent:* I think it—probably just, building confidence that we talk about.	*Confidence* is in resource, with *self-esteem.* No changes made.	0
• *Child:* I'm just confused of what joints are?	Remove *joints* from resource.	1
• *Interviewer: D*o you know what anxiety and depression is?<br>*Child:* Depression means you're very sad and gloomy.<br>*Interviewer:* Sad and gloomy. That's great. Anxiety is—<br>*Child:* It's when you get really stressed out and stuff.<br>*Child:* Stressed out and you don't know how to calm down.	Amended resource to replace some hard words or added to *hard words* section. Children showed very variable ability to read and understand some words but resources are for families to read together. Thus, left some words e.g. *self-esteem,* with description in *hard words* section to support parents to provide explanations	1
• *Child:* It's working memory. Working memory actually mean you remember stuff?<br>*Child:* I feel like that memory is just remembering. It has nothing to do with sport.	Changed *aids working memory* to *helps you remember things better.*	1
• *Interviewer:* Are there any words that you think might be tricky for children—<br>*Child:* Self-esteem and relaxing and confidence.<br>*Child:* Self-esteem, it's controlling yourself when you get angry.	*Self-esteem, relax and confidence* explanations in *hard words* section.	3
Researchers’ observations	Action	Changes to resource
• Observed that families found that there was a lot of information on this page. Was there a way to simplify or group the information better to aid reading and understanding?	Reviewed information to see if there was a way of grouping the information better. Grouped information into four sections: *physical, mood, brain* and *social gains.*	1
**Total number of changes to resource = 34**<br>**Number of child and parent co-designers’ suggestions that led to changes = 30**		
